# Effects of Bracing Combined With Tele‐Rehabilitation‐Guided Family Physiotherapeutic Scoliosis‐Specific Exercises on Adolescent Idiopathic Scoliosis

**DOI:** 10.1111/os.70265

**Published:** 2026-01-30

**Authors:** Tao Chen, Hao Zhou, Qizhu Chen, Linjie Chen, Zhiguang Zhang, Zhendi Shu, Songhe Jiang, Xiangyang Wang, Aimin Wu, Xiaoli Huang

**Affiliations:** ^1^ Department of Orthopaedics, Key Laboratory of Structural Malformations in Children of Zhejiang Province, Key Laboratory of Orthopaedics of Zhejiang Province The Second Affiliated Hospital and Yuying Children's Hospital of Wenzhou Medical University Wenzhou Zhejiang China

**Keywords:** adolescent idiopathic scoliosis, angle of trunk rotation, Chêneau brace, Cobb angle, exercise, rehabilitation

## Abstract

**Objective:**

Adolescent idiopathic scoliosis (AIS) necessitates multimodal management strategies integrating orthotic intervention and physiotherapeutic scoliosis‐specific exercises (PSSE). This study aimed to compare the clinical efficacy of brace therapy combined with tele‐rehabilitation‐guided PSSE versus brace treatment with self‐guided home‐based PSSE in mitigating spinal deformity progression.

**Methods:**

A cohort of 67 treatment‐naïve AIS patients from a tertiary scoliosis center (July 2021–July 2023) was stratified into two intervention groups: (1) tele‐rehabilitation (real‐time digitally supervised PSSE) and (2) autonomous practice (self‐guided home PSSE). Longitudinal evaluations at baseline, 6, 12, and 24‐month intervals included radiographic Cobb angle quantification, scoliometric angle of trunk rotation (ATR) assessment, and Scoliosis Research Society‐22 (SRS‐22) patient‐reported outcomes. Treatment success was categorized as improvement (Cobb reduction ≥ 5°), stability (change < 5°), or progression (increase ≥ 5°). Data were analyzed using paired and independent *t*‐tests, Mann–Whitney *U* test, and Pearson's χ^2^ test.

**Results:**

At 24‐month follow‐up, the tele‐rehabilitation group exhibited significantly higher Cobb angle improvement rates (70.6% vs. 57.6%, *p* < 0.05) and lower progression rates (2.9% vs. 6.1%) compared to the autonomous practice group. Axial rotation correction demonstrated superior outcomes in the supervised cohort (final ATR: 6.9° ± 1.9° vs. baseline, *p* < 0.01). All SRS‐22 domains showed clinically meaningful improvements (*p* < 0.05).

**Conclusion:**

Tele‐rehabilitation‐guided PSSE combined with bracing demonstrates enhanced efficacy over self‐guided protocols in achieving three‐dimensional deformity correction, stabilizing curve progression, and optimizing patient‐centered outcomes. Structured digital supervision emerges as a critical adjunct to orthotic management, advocating for technology‐integrated conservative strategies in adolescent spinal deformity care.

## Graphical abstract

For adolescents with idiopathic scoliosis, tele‐rehabilitation‐guided physiotherapeutic scoliosis‐specific exercises combined with the Chêneau brace can effectively improve the degree of spinal deformity. This new strategy may be a reliable and efficient conservative treatment.

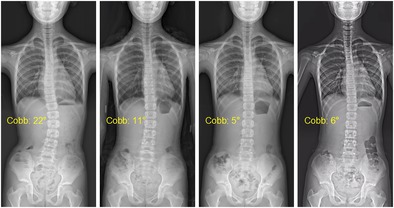

## 1 Introduction

Adolescent idiopathic scoliosis (AIS) is a three‐dimensional spinal deformity characterized by coronal plane curvature, vertebral rotation, and sagittal imbalance, with severe cases leading to functional impairments and psychosocial burdens [1]. The risk of AIS progression is closely related to the initial scoliosis angle and growth potential [2]. Therefore, to prevent the exacerbation of scoliosis as much as possible and reduce the surgical rate, early detection, intervention, and treatment of AIS patients with immature growth and large scoliosis angles are very important.

Braces are primarily used to prevent the worsening of the deformity, or to correct part of the deformity during periods of growth potential. They aim to improve physical appearance, control pain, and potentially avoid the need for surgical intervention [3, 4]. The Chêneau brace, commonly used to treat scoliosis in China, possesses both active and passive mechanisms. Apart from the passive corrective mechanisms involving multiple three‐point forces, its active corrective mechanisms include gravity resistance, growth guidance, muscle activity, and respiration [5, 6]. Research has shown that the Chêneau brace can potentially enhance comfort, compliance, and curve correction, which are advantageous for reversing the vicious cycle [7, 8].

However, AIS correction is a relatively lengthy process, involving skeletal growth and development related to appropriate exercises [9, 10]. Physiotherapeutic scoliosis‐specific exercises (PSSE) are one of the conservative treatments recommended by the Society on Scoliosis Orthopedic and Rehabilitation Treatment (SOSORT) [11, 12]. Reportedly, specific movements during PSSE could improve posture and achieve more symmetrical paravertebral muscle activity, demonstrating potential value [13]. This aligned with the corrective mechanisms of the Chêneau brace.

Conservative management prioritizes early intervention during skeletal immaturity, leveraging bracing to modulate curve progression and PSSE to optimize neuromuscular function [3, 12]. The Chêneau brace, widely implemented in clinical practice, combines passive three‐point corrective forces with active mechanisms promoting postural alignment and respiratory capacity [7]. When integrated with PSSE, this approach aims to enhance postural symmetry and paravertebral muscle coordination, potentially interrupting the biomechanical cascade of deformity progression [14].

Despite the accelerated adoption of tele‐rehabilitation during the pandemic, a critical evidence gap persists regarding its long‐term efficacy in AIS conservative management. Specifically, the capacity of real‐time digitally supervised PSSE combined with Chêneau bracing to modulate three‐dimensional deformity progression (compared to self‐guided regimens) remains unvalidated, particularly in terms of sustained biomechanical correction and neuromuscular adaptation during adolescence. This study aims to (i) compare the 24‐month radiographic and functional outcomes of brace therapy combined with tele‐rehabilitation versus self‐guided home exercises in treatment‐naïve AIS patients; (ii) establish an evidence base for technology‐integrated conservative strategies to optimize AIS management.

## 2 Methods

### 2.1 Study Design and Participants

This cohort study consecutively enrolled 74 treatment‐naïve patients with adolescent idiopathic scoliosis (AIS) from a tertiary spinal center at the Second Affiliated Hospital and Yuying Children's Hospital of Wenzhou Medical University between July 2021 and July 2023. The institutional review board approved the protocol (No. LCKY2020‐295), and written informed consent was obtained from all participants and guardians. Eligible patients met the following criteria: (1) Scoliosis Research Society (SRS)‐defined AIS diagnosis confirmed through clinical and radiographic evaluation; (2) skeletal immaturity (Risser grade ≤ 2; age 10–15 years); (3) moderate coronal deformity (Cobb angle 20°–45°). Exclusion criteria included nonidiopathic etiology, prior orthotic treatment, cognitive impairment, or premature trial discontinuation. Following standardized counseling on conservative management, participants were stratified by baseline Cobb angle and skeletal maturity into two cohorts: Tele‐rehabilitation group (*n* = 34): Real‐time digitally supervised PSSE and Autonomous practice group (*n* = 33): Self‐guided home‐based PSSE.

### 2.2 Interventions

#### 2.2.1 Bracing Protocol

All participants received computer‐aided, individualized Chêneau braces manufactured through a computer‐aided workflow. (1) Digital Modeling: Integration of full‐spine radiographs and surface topography scans using CAD/CAM software (Materialize Mimics) to map anatomical pressure zones (primarily apical vertebrae and rib hump) with design targets of 20–30 mmHg (targeted for optimal correction force without tissue compromise). The core correction standards for the brace were defined as follows: (i) Coronal plane correction: a primary precision target of ≥ 50% in‐brace correction rate for the maximum Cobb angle, aiming to halt curve progression and achieve clinically meaningful deformity improvement; (ii) Vertebral rotation correction: reduction or prevention of thoracic rotation and “rib hump” deformity progression via customized pressure pad design; (iii) Sagittal plane maintenance: preservation of normal physiological curves, with thoracic kyphosis and lumbar lordosis maintained within age‐matched normal ranges to avoid iatrogenic complications (e.g., flatback deformity, insufficient kyphosis) from overcorrection of coronal curvature. (2) CNC Fabrication: Braces were milled from 4 mm thick high‐temperature polyethylene thermoplastic sheets. (3) Adaptation & Wear: An adaptation phase utilized a gradual wear schedule: nocturnal use only (Weeks 1 and 2), increasing to > 22 h/day by month 1. Monthly adjustments by spinal surgeons and physiotherapists ensured optimal three‐dimensional correction based on patient‐specific biomechanics, adhering to SOSORT guidelines with a focus on pressure point relief and curve correction maintenance. A representative case example of the CAD‐designed Chêneau brace is provided in Figure S1.

#### 2.2.2 PSSE Protocol Essentials

**FIGURE 1:**
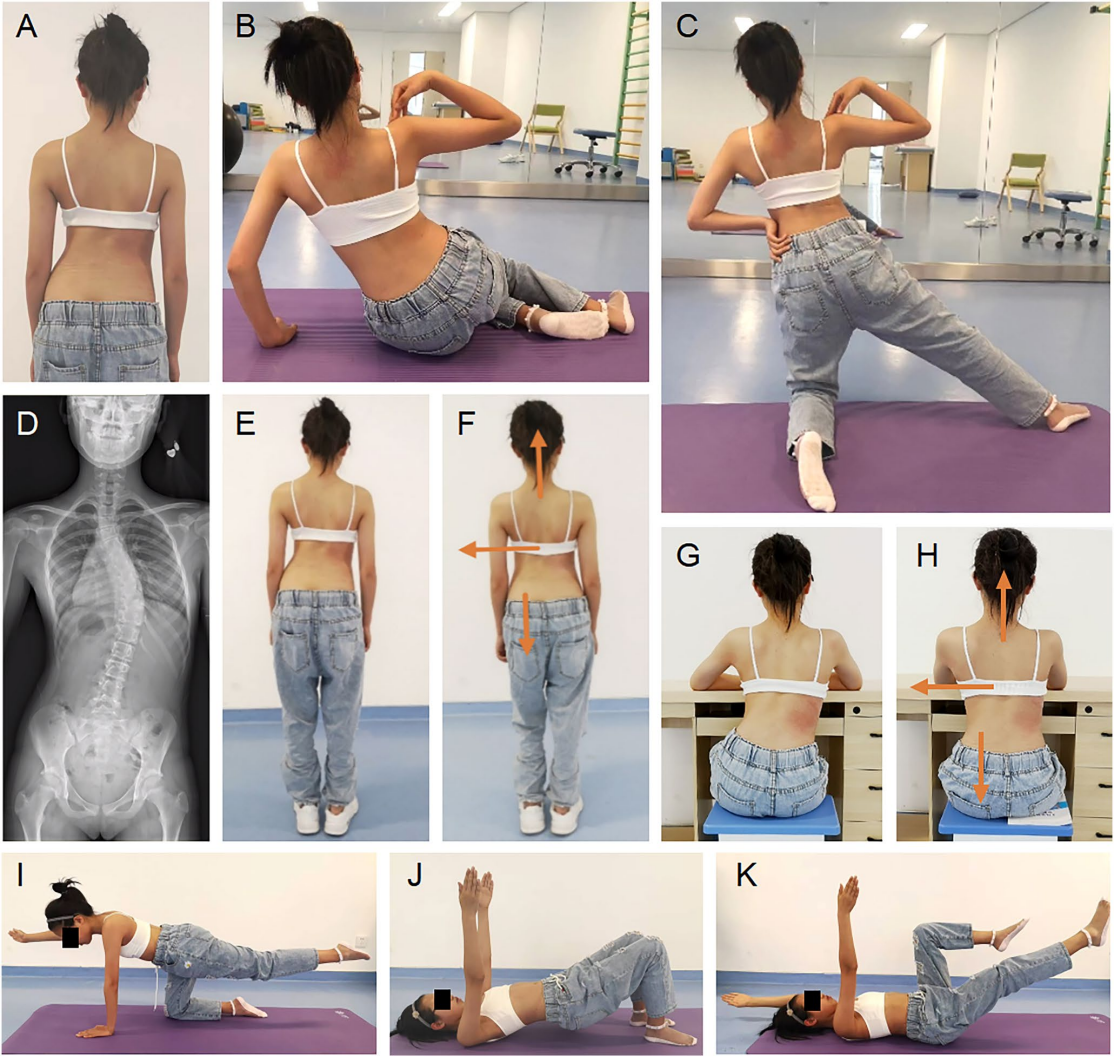
Individualized treatment for patients with different classifications of scoliosis. (A) Morphological image pretreatment. (B, C) Examples of corrective exercises for improving lateral curvature in the frontal plane and rotation in the horizontal plane. These include lowering the left pelvic side, shifting the thoracolumbar curve to the left, expanding the concave left side of the thoracolumbar area upon inhalation, and isometrically contracting the back muscles upon exhalation. Exercise parameters: Maintain forced exhalation for 5–10 s per breath, with 5 breaths as one set; 10 sets per exercise session. (D) X‐ray image pretreatment. (E) Habitual standing posture. (F) Corrective standing posture. (G) Habitual sitting posture. (H) Corrective sitting posture. (I–K) Core strengthening exercises for improving spine stability. Exercise parameters: Hold each posture for 5–10 s per repetition, with 10 repetitions as one set; three sets per exercise session. The specific exercises (B, C, I–K) were performed without wearing the Chêneau brace, while the daily standing and sitting postures (E–H) were maintained with the Chêneau brace worn.

Standardized and individualized PSSE treatment plans were designed for each patient, including: (1) Postural Adjustment for Activities of Daily Living: Integration of corrective posture into routine activities such as standing, sitting, and walking (2) Corrective Exercises: using the Schroth method (3) Exercises for Normalizing Sagittal Plane Deformities (4) Core Strengthening Exercises (Figure 1).

#### 2.2.3 Implementation

Tele‐rehabilitation group: Synchronous 40‐min sessions (5×/week) via Tencent Meeting (meeting.tencent.com). Therapists visually monitored patients' exercise postures and spinal alignment in real time via video. Kinematic errors (e.g., inadequate rotation, improper alignment) were detected through direct observation, with immediate corrective feedback provided via verbal guidance or live demonstration.

Autonomous practice group: Guardian‐supervised home exercises (≥ 200 min/week) using illustrated manuals. Guardians completed competency‐based training (Figure S2: ≥ 4/5 pain recognition, < 5° angle error) and documented adherence via standardized exercise logs (Figure S3: exercise categories, parameters, completion status, signature).

Adherence Reinforcement: Digital attendance tracking with automated reminders.

**FIGURE 2:**
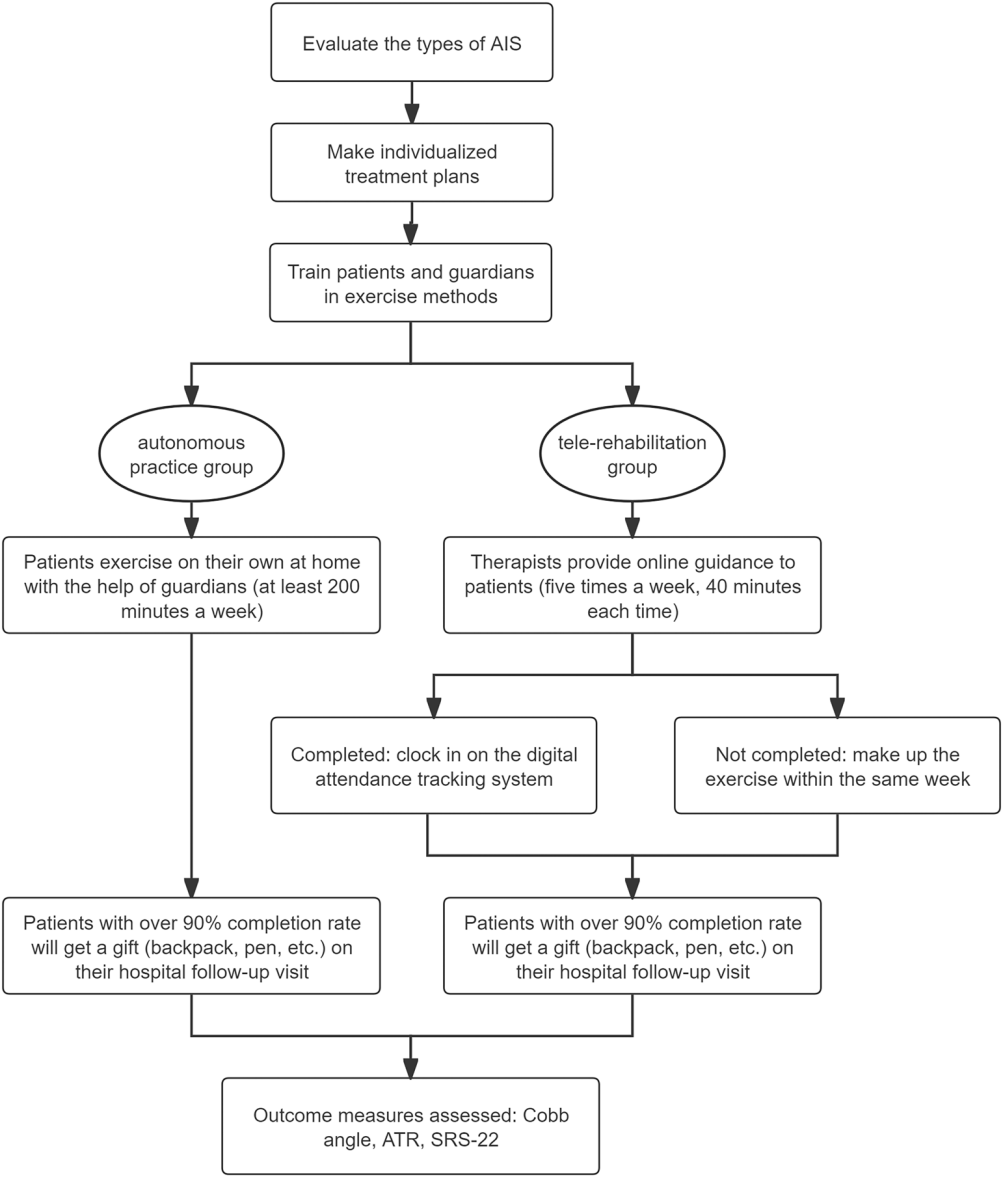
Flow diagram of the patient's exercise program.

Incentivization protocol: ≥ 90% adherence rewarded with educational materials (Figure 2).

### 2.3 Outcome Measures

#### 2.3.1 Primary Endpoints

##### 2.3.1.1 Coronal Correction

Maximum Cobb angle measured on standing full‐spine radiographs 24 h post‐brace removal categorized as improvement (≥ 5° reduction), stability (< 5° change), or progression (≥ 5° increase).

##### 2.3.1.2 Axial Rotation

Angle of trunk rotation (ATR) quantified via standardized Adams forward bend test using Scoliometer (Orthopedic Systems Inc.).

#### 2.3.2 Secondary Endpoints

##### 2.3.2.1 Quality of Life

Validated Chinese SRS‐22 questionnaire assessing five domains: function (five items), Pain (five items), Self‐Image (five items), Mental Health (five items), and Satisfaction with Management (two items). Each item is scored 1 (worst) to 5 (best). Domain scores are the average of items within that domain (range 1–5). A total score (average of all 22 items, range 1–5) provides an overall measure of health‐related quality of life.

#### 2.3.3 Assessment Protocol

Dual‐blinded measurements by senior spinal surgeons at baseline, 6, 12, and 24‐month intervals. Radiographic analysis using semiautomated edge detection software (Surgimap Spine). Intraclass correlation coefficients > 0.85 for all measurement tools.

### 2.4 Statistical Analysis

Statistical analyses were conducted using SPSS version 26.0 (IBM Corp). A post hoc power analysis confirmed 82% power to detect ≥ 5° Cobb angle differences (effect size *d* = 0.53), consistent with prior PSSE trials. Continuous variables were expressed as mean ± standard deviation (SD) if normally distributed, or median (interquartile range) otherwise. Categorical variables were reported as frequencies (percentages). Normality was assessed using the Kolmogorov–Smirnov test (α = 0.05). Normally distributed continuous variables were analyzed with parametric tests (paired *t*‐test for intragroup comparisons; independent *t*‐test for intergroup comparisons). Non‐normally distributed continuous variables were analyzed with the Mann–Whitney *U* test. Categorical variables were compared using Pearson's χ^2^ test with Yates' continuity correction. All tests were two‐tailed, with statistical significance set at *p* < 0.05.

## 3 Results

### 3.1 Participant Allocation and Baseline Characteristics

Following eligibility screening, 33 patients were allocated to the autonomous practice cohort and 34 to the tele‐rehabilitation cohort. Baseline demographic and clinical characteristics—including sex, curve pattern, age, anthropometric measures (height, weight, BMI), radiographic parameters (maximum Cobb angle, ATR), and skeletal maturity (Risser grade)—demonstrated no statistically significant intergroup differences (*p* > 0.05 for all comparisons; Table 1).

**TABLE 1:** The baseline characteristics of the included patients in the two groups.

	Autonomous practice	Tele‐rehabilitation	MD (95% CI)	*p*
Sex (Male/Female)	12/21	8/26	—	0.251
Curve pattern, *n* (%)				0.850
Thoracic	16 (48.5)	18 (52.9)		
Thoracolumbar	10 (30.3)	9 (26.5)		
Lumbar	6 (18.2)	5 (14.7)		
Double major	1 (3.0)	2 (5.9)		
Age, years	12.2 (1.6)	12.9 (1.8)	−0.7 (−1.5 to 0.2)	0.118
Height, m	1.5 (0.1)	1.5 (0.1)	0.0 (0.0 to 0.1)	0.861
Weight, kg	38.5 (6.4)	38.6 (6.4)	−0.2 (−3.3 to 2.9)	0.902
Body mass index, kg/m^2^	16.6 (2.7)	16.8 (2.5)	−0.2 (−1.4 to 1.1)	0.787
Risser grade	1.2 (0.9)	1.3 (0.8)	−0.1 (−0.5 to 0.3)	0.608
Maximum Cobb angle, °	28.9 (6.3)	28.6 (5.6)	0.4 (−2.6 to 3.3)	0.811
Angle of trunk rotation, °	10.7 (3.2)	10.9 (2.8)	−0.2 (−1.6 to 1.3)	0.832

*Note*: Values are expressed as mean (standard deviation) unless specified. Curve pattern comparison by χ^2^ test (χ^2^ = 0.81, *p* = 0.85).

Abbreviations: CI, confidence interval; MD, mean difference.

### 3.2 Compliance Monitoring

Adherence to brace wear and PSSE was monitored throughout the 24‐month follow‐up period. As detailed in Table 2, participants in the tele‐rehabilitation group demonstrated significantly higher compliance compared to the autonomous practice group.

1. Brace Adherence: The tele‐rehabilitation group achieved a significantly higher mean daily brace wear time (18.7 ± 1.3 h vs. 16.2 ± 2.1 h, *p* < 0.001) and a significantly greater proportion met the target compliance threshold of ≥ 18 h/day (88.2% vs. 51.5%, *p* = 0.001).
2. PSSE Adherence: Similarly, exercise adherence was significantly superior in the tele‐rehabilitation group, evidenced by a higher mean session completion rate (92.6% ± 5.9% vs. 74.3% ± 12.8%, *p* < 0.001) and a greater proportion achieving the ≥ 80% compliance target (97.1% vs. 60.6%, *p* < 0.001).
3. Discontinuations: While overall dropout rates (tele‐rehabilitation: 5.9%, autonomous: 15.2%, *p* = 0.261) and technical dropout rates (tele‐rehabilitation: 2.9%, autonomous: 9.1%, *p* = 0.351) were lower in the tele‐rehabilitation group, these differences did not reach statistical significance.

**TABLE 2:** Compliance monitoring outcomes at 24‐month follow‐up.

	Autonomous practice	Tele‐rehabilitation	*p*
Brace adherence			
Mean wear time, hour/day (SD)	16.2 (2.1)	18.7 (1.3)	0.000
≥ 18 hours/day compliance, *n* (%)	17 (51.5)	30 (88.2)	0.001
PSSE adherence			
Mean session completion, % (SD)	74.3 (12.8)	92.6 (5.9)	0.000
≥ 80% compliance rate, *n* (%)	20 (60.6)	33 (97.1)	0.000
Discontinuations			
Technical dropouts, *n* (%)	3 (9.1)	1 (2.9)	0.351
Overall dropout rate, *n* (%)	5 (15.2)	2 (5.9)	0.261

Abbreviations: PSSE, physiotherapeutic scoliosis‐specific exercises; SD, standard deviation.

### 3.3 Coronal Plane Correction Outcomes

**FIGURE 3:**
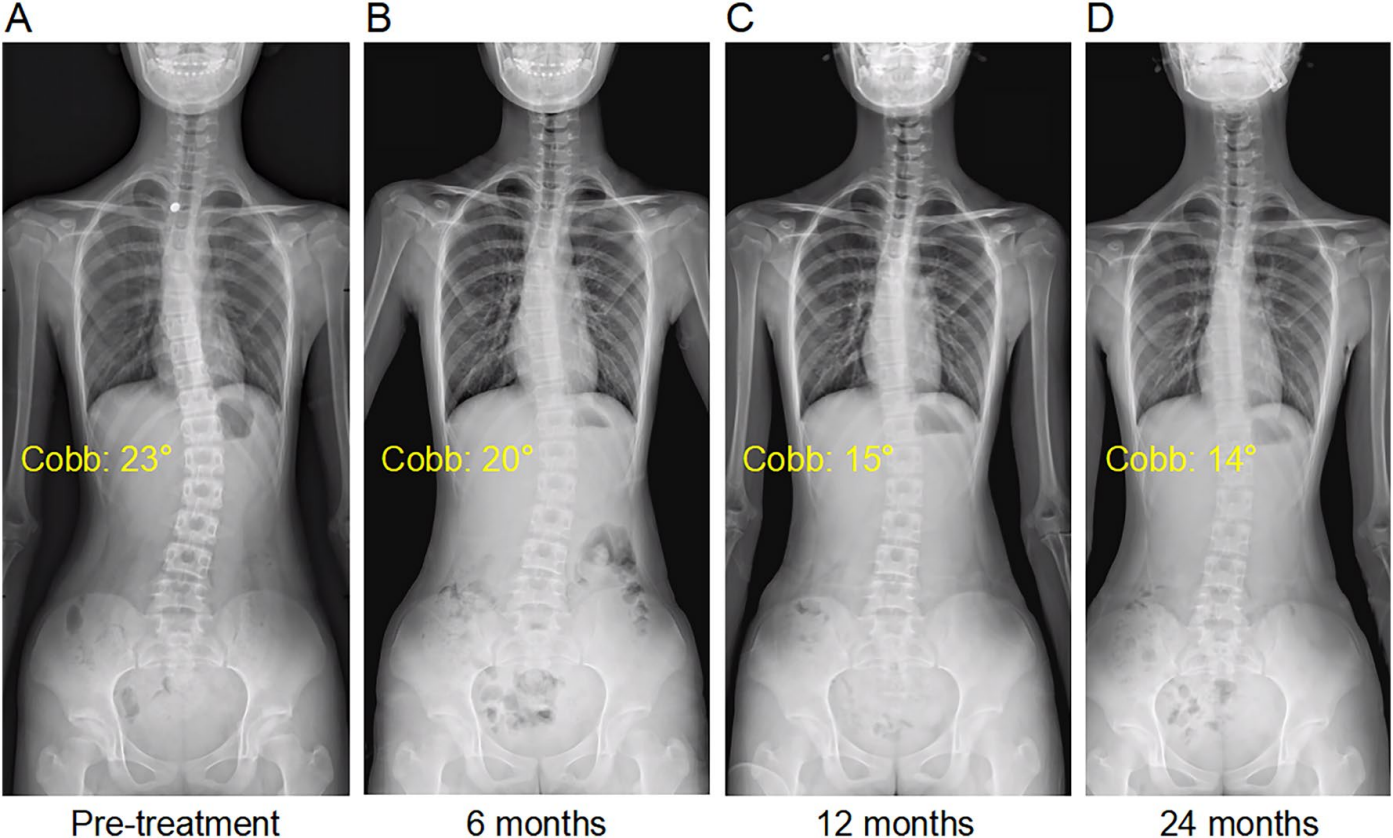
A typical case in the autonomous practice group. A 12‐year‐old male patient with adolescent idiopathic scoliosis (height: 1.54 m, weight: 47 kg, BMI: 19.82 kg/m^2^). (A–D) X‐ray changes from pretreatment to 24 months of follow‐up showed a decrease in the maximum Cobb angle from 23° to 14°.

**FIGURE 4:**
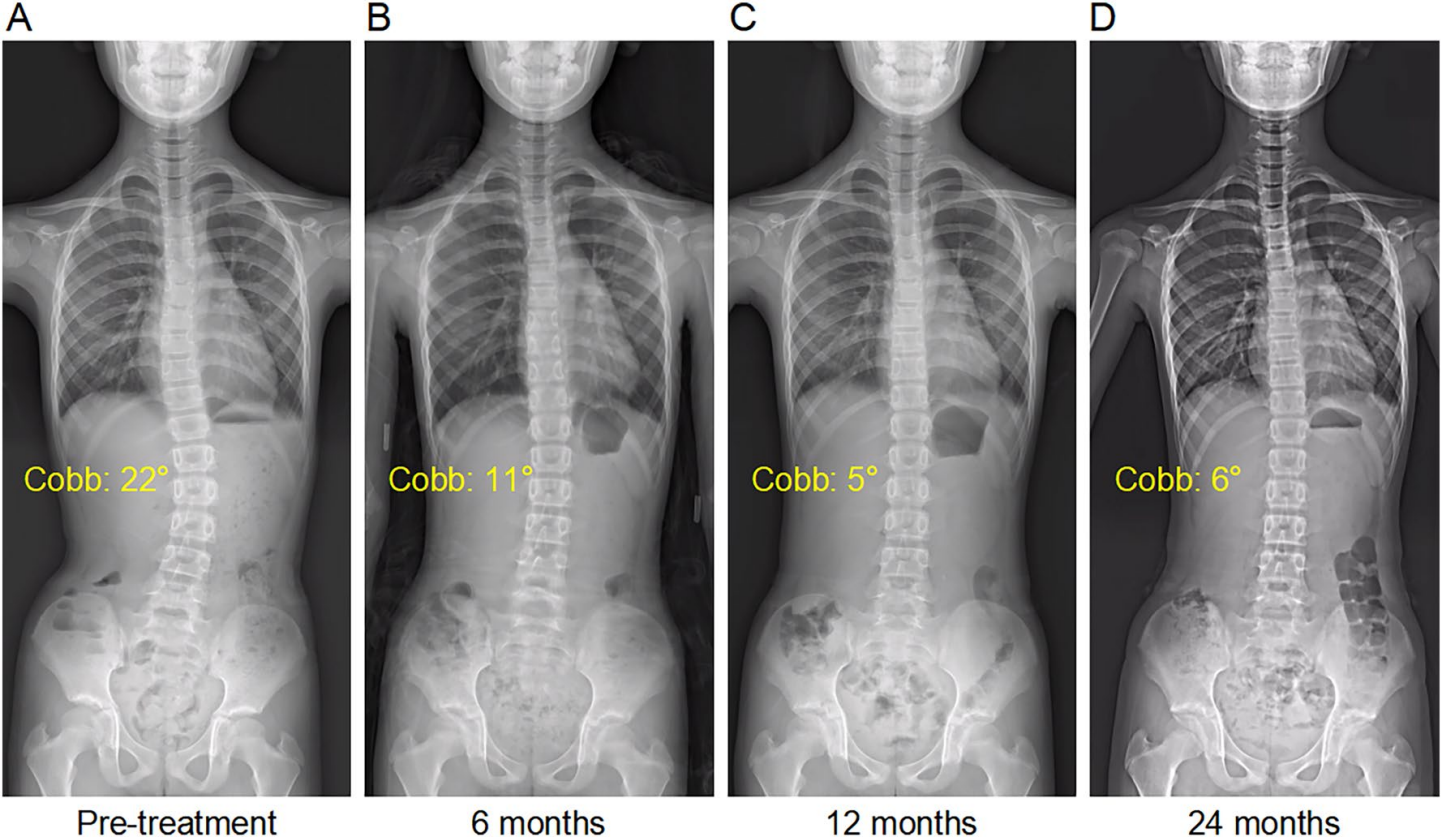
A typical case in the tele‐rehabilitation group. An 11‐year‐old female patient with adolescent idiopathic scoliosis (height: 1.42 m, weight: 30 kg, BMI: 14.88 kg/m^2^). (A–D) X‐ray changes from pretreatment to 24 months of follow‐up showed a decrease in the maximum Cobb angle from 22° to 6°.

Both cohorts exhibited progressive Cobb angle reductions from baseline through 24‐month follow‐up (Figures 3 and 4). In the autonomous practice group, the mean maximum Cobb angle decreased from 28.9° ± 6.3° to 25.4° ± 6.6° (*p* < 0.01), with 57.6% (19/33) achieving ≥ 5° improvement, 36.4% (12/33) stabilizing, and 6.1% (2/33) progressing at final follow‐up.

**FIGURE 5:**
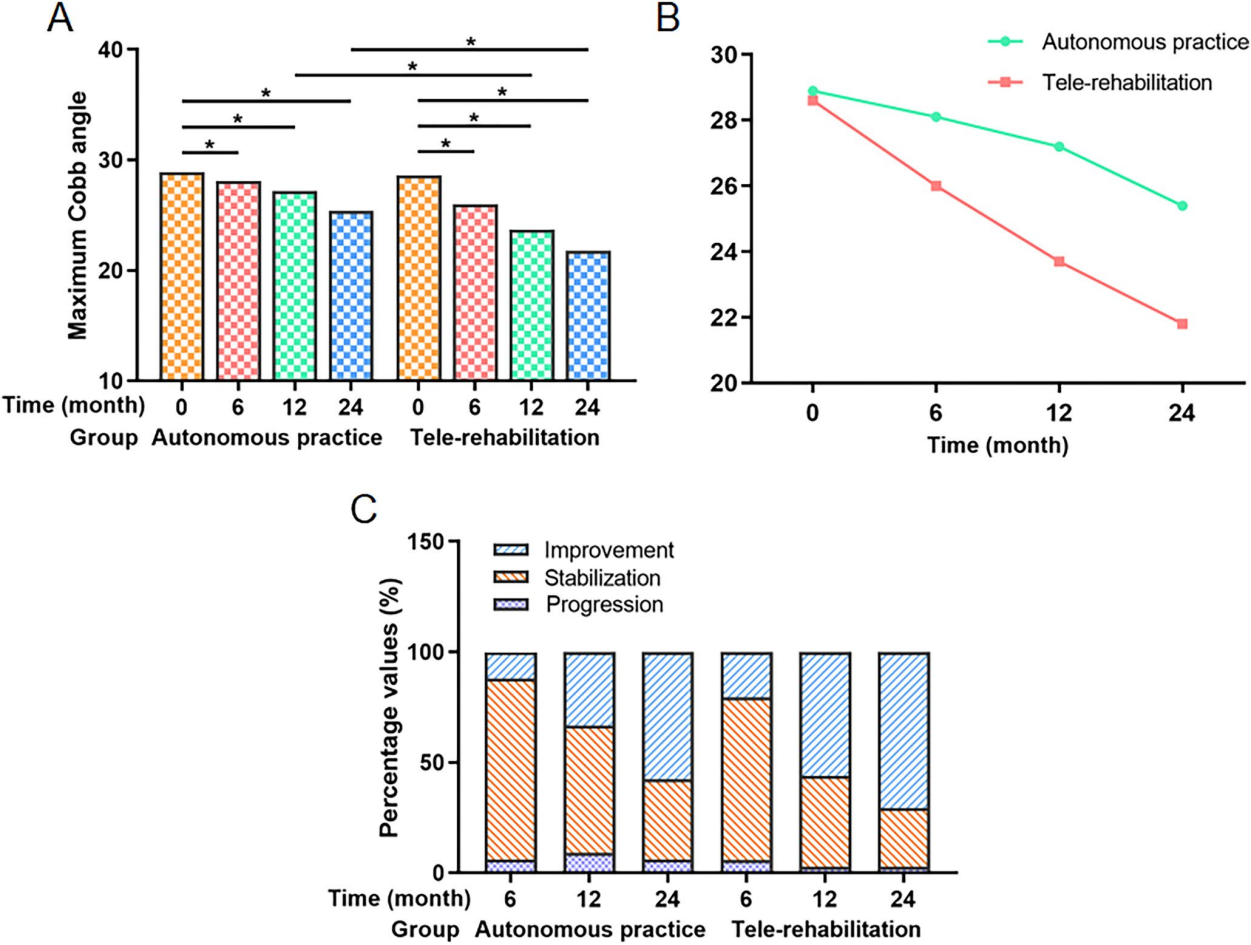
Comparison of maximum Cobb angle. (A) Intra‐ and intergroup comparison of maximum Cobb angle in the autonomous practice and the tele‐rehabilitation groups. (B) The maximum Cobb angle change curve graph of the autonomous practice and the tele‐rehabilitation groups. (C) Numbers and percentage values of improvement, stabilization, and progression in maximum Cobb angle. * Denotes a *p* value below 0.05.

The tele‐rehabilitation cohort demonstrated superior coronal correction, achieving a final mean Cobb angle of 21.8° ± 5.8 (*p* < 0.01), with 70.6% (24/34) showing improvement, 26.5% (9/34) stabilization, and 2.9% (1/34) progression. Intergroup differences reached statistical significance at 12 months (*p* = 0.029) and 24 months (*p* = 0.021) postintervention (Tables 3 and 4, Figure 5).

**TABLE 3:** Maximum Cobb angle comparison (°).

	Autonomous practice			Tele‐rehabilitation			*p*
	Mean (SD)	Range	*p*	Mean (SD)	Range	*p*	
0 month	28.9 (6.3)	20–40	—	28.6 (5.6)	20–37	—	0.811
6 months	28.1 (5.9)	19–38	0.001*	26.0 (5.7)	11–35	0.000*	0.151
12 months	27.2 (6.4)	15–42	0.001*	23.7 (6.1)	5–33	0.000*	0.029*
24 months	25.4 (6.6)	14–43	0.000*	21.8 (5.8)	6–32	0.000*	0.021*

Abbreviation: SD, standard deviation.

* *p* value below 0.05.

**TABLE 4:** Numbers and percentage values of progression, stabilization, and improvement in maximum Cobb angle.

	Progression		Stabilization		Improvement	
	*n*	Percentage	*n*	Percentage	*n*	Percentage
Autonomous practice						
6 months	2	6.1	27	81.8	4	12.1
12 months	3	9.1	19	57.6	11	33.3
24 months	2	6.1	12	36.4	19	57.6
Tele‐rehabilitation						
6 months	2	5.9	25	73.5	7	20.6
12 months	1	2.9	14	41.2	19	55.9
24 months	1	2.9	9	26.5	24	70.6

To address potential confounders of exercise scheduling, a subgroup analysis compared tele‐rehabilitation (structured 5 × 40‐min/week, *n* = 34) with high frequency autonomous practice (≥ 4 sessions/week, total 200‐min/week, *n* = 20). Despite matched duration and frequency (≥ 4 sessions/week), the tele‐rehabilitation group achieved significantly greater Cobb angle reduction at 24 months (−6.8° ± 2.1° vs. −4.6° ± 1.9°, *p* < 0.001) and a higher rate of clinically meaningful improvement (≥ 5° reduction: 70.6% vs. 55.0%, *p* = 0.038) (Table 5). This underscores that real‐time supervision—beyond mere frequency—optimizes biomechanical precision (e.g., rotational breathing control, asymmetric muscle activation).

**TABLE 5:** 24‐month Cobb angle outcomes comparison between Tele‐rehabilitation and high frequency Autonomous practice groups.

	Tele‐rehabilitation (*n* = 34)	High frequency autonomous practice (≥ 4 sessions/week, *n* = 20)	*p*
Baseline Cobb angle (°)	28.6 ± 5.6	28.7 ± 5.9	0.943
24‐month Cobb angle (°)	21.8 ± 5.8	24.1 ± 6.2	0.021
△Cobb angle (°)	−6.8 ± 2.1	−4.6 ± 1.9	0.000
Improvement rate (≥ 5°), *n* (%)	24 (70.6%)	11 (55.0%)	0.038

### 3.4 Axial Rotation Improvements

**FIGURE 6:**
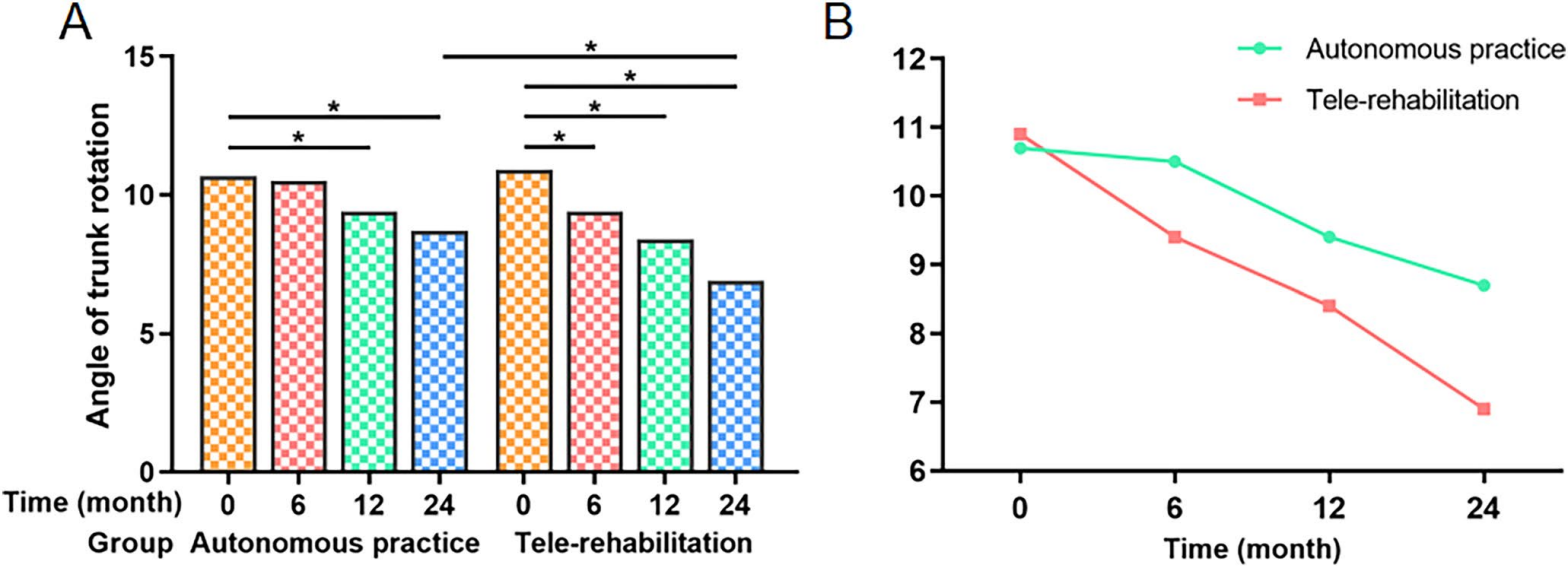
Comparison of ATR. (A) Intra‐ and intergroup comparison of ATR in the autonomous practice and the tele‐rehabilitation groups. (B) The ATR change curve graph of the autonomous practice and the tele‐rehabilitation groups. * Denotes a *p* value below 0.05.

Baseline ATR measurements showed no intergroup disparity (*p* = 0.832). At 24‐month follow‐up, the autonomous practice cohort achieved moderate ATR reduction (10.7° ± 3.2° to 8.7° ± 2.9°, *p* < 0.001), while the tele‐rehabilitation group demonstrated superior correction (10.9° ± 2.8° to 6.9° ± 1.9°, *p* < 0.001). Significant intergroup differences emerged at the final assessment (*p* = 0.005; Table 6, Figure 6). A sensitivity analysis comparing tele‐rehabilitation participants who received gifts (adherence ≥ 90%, *n* = 31) vs. non‐recipients (*n* = 3) showed no significant outcome differences: Cobb angle change: 6.9° ± 1.8° vs. 6.7° ± 2.1° (*p* = 0.82); ATR reduction: 4.1° ± 1.2° vs. 3.9° ± 1.4° (*p* = 0.79). This suggests that incentives did not disproportionately influence clinical outcomes.

**TABLE 6:** Angle of trunk rotation comparison.

	Autonomous practice			Tele‐rehabilitation			*p*
	Mean (SD)	Range (°)	*p*	Mean (SD)	Range (°)	*p*	
0 month	10.7 (3.2)	5–15	—	10.9 (2.8)	5–15	—	0.832
6 months	10.5 (3.1)	5–16	0.103	9.4 (2.4)	5–14	0.000*	0.121
12 months	9.4 (3.0)	5–16	0.000*	8.4 (2.2)	4–13	0.000*	0.131
24 months	8.7 (2.9)	5–16	0.000*	6.9 (1.9)	4–12	0.000*	0.005*

Abbreviation: SD, standard deviation.

* *p* value below 0.05.

### 3.5 Patient‐Reported Outcomes

SRS‐22 scores demonstrated progressive improvements in both cohorts (Tables 7 and 8). The autonomous practice group showed significant baseline‐to‐24‐month improvements in five domains. The tele‐rehabilitation cohort exhibited earlier significant enhancements with function and treatment satisfaction domains, showing clinically meaningful improvements by 12 months (*p* < 0.05 for all). At 24 months, all SRS‐22 domains achieved statistical significance versus baseline in both cohorts (*p* < 0.05).

**TABLE 7:** SRS‐22 scores in the autonomous practice group.

	0 month		6 months		12 months		24 months	
	Mean (SD)	*p*	Mean (SD)	*p*	Mean (SD)	*p*	Mean (SD)	*p*
Function	4.3 (0.3)	—	4.3 (0.3)	0.667	4.4 (0.3)	0.084	4.4 (0.3)	0.005*
Pain	4.2 (0.3)	—	4.3 (0.3)	0.319	4.4 (0.2)	0.024*	4.4 (0.3)	0.002*
Self‐image	4.3 (0.4)	—	4.4 (0.4)	0.141	4.4 (0.3)	0.114	4.5 (0.3)	0.023*
Mental health	4.3 (0.4)	—	4.3 (0.3)	0.630	4.4 (0.3)	0.025*	4.5 (0.3)	0.004*
Satisfaction with management	4.1 (0.5)	—	4.2 (0.5)	0.296	4.3 (0.3)	0.083	4.4 (0.4)	0.020*

Abbreviation: SD, standard deviation.

* *p* value below 0.05.

**TABLE 8:** SRS‐22 scores in the tele‐rehabilitation group.

	0 month		6 months		12 months		24 months	
	Mean (SD)	*p*	Mean (SD)	*p*	Mean (SD)	*p*	Mean (SD)	*p*
Function	4.2 (0.3)	—	4.2 (0.3)	0.501	4.3 (0.3)	0.035*	4.3 (0.3)	0.000*
Pain	4.1 (0.3)	—	4.2 (0.3)	0.200	4.2 (0.2)	0.024*	4.3 (0.3)	0.001*
Self‐image	4.3 (0.4)	—	4.3 (0.3)	0.668	4.3 (0.3)	0.136	4.5 (0.2)	0.002*
Mental health	4.2 (0.4)	—	4.3 (0.3)	0.463	4.4 (0.2)	0.002*	4.5 (0.2)	0.000*
Satisfaction with management	4.1 (0.4)	—	4.1 (0.4)	0.856	4.3 (0.3)	0.024*	4.5 (0.4)	0.000*

Abbreviation: SD, standard deviation.

* *p* value below 0.05.

## 4 Discussion

The findings of this cohort study demonstrate that tele‐rehabilitation‐guided physiotherapeutic scoliosis‐specific exercises (PSSE) combined with Chêneau bracing yield significantly superior three‐dimensional deformity correction and patient‐reported outcomes compared to self‐guided home exercise protocols in adolescents with idiopathic scoliosis. At the critical 24‐month follow‐up—spanning peak adolescent growth periods where curve progression risk is highest—the tele‐rehabilitation group achieved not only statistically significant but clinically meaningful advantages: a near doubling of Cobb angle reduction (Δ6.8° vs. Δ3.5°; *p* < 0.01), representing a greater corrective magnitude. This was paralleled by a 22.6% relative increase in patients achieving clinically meaningful improvement (70.6% vs. 57.6%), potentially sparing additional adolescents from surgical intervention.

Critically, the tele‐rehabilitation cohort demonstrated superiority in axial derotation (ATR reduction Δ4.0° vs. Δ2.0°; *p* < 0.01), suggesting real‐time kinematic feedback uniquely optimizes rotational correction mechanics that passive bracing alone cannot achieve. These radiographic improvements translated into functionally relevant outcomes: significantly earlier (3‐month vs. 6‐month) and more substantial gains in SRS‐22 domains, particularly physical function (Δ0.8 vs. Δ0.3; *p* < 0.001) and treatment satisfaction (Δ1.2 vs. Δ0.6; *p* < 0.001), indicating patients perceived tangible benefits from enhanced exercise precision.

Collectively, these results address three critical evidence gaps in spinal deformity management: (1) They confirm digital supervision's capacity to sustain exercise fidelity over adolescence's high‐risk growth period, (2) They demonstrate that tele‐rehabilitation synergistically enhances brace mechanics to achieve true 3D correction beyond the coronal plane, and (3) They establish a causal pathway from technical precision (via real‐time feedback) → biomechanical efficacy (Cobb/ATR improvement) → patient‐perceived benefit (SRS‐22 gains). This advances tele‐rehabilitation from a pandemic contingency to a high‐precision therapeutic strategy with potential to redefine conservative scoliosis management paradigms.

### 4.1 Integration With Existing Evidence

Our observed 70.6% Cobb angle improvement rate in the tele‐rehabilitation cohort aligns with—and extends—prior research on supervised PSSE efficacy. A 2020 multicenter trial by Schreiber et al. reported 65% curve stabilization rates with therapist‐guided Schroth exercises at 12 months, though our 24‐month outcomes demonstrate significantly greater durability of correction [15]. Our 24‐month outcomes substantiate SOSORT guidelines emphasizing the importance of structured exercise supervision, particularly during peak growth velocity phases where biomechanical precision is critical [1]. The significant between‐group differences in Cobb angle reduction (Δ3.6°, *p* < 0.05) and axial rotation correction (Δ1.8°, *p* < 0.01) at final follow‐up suggest that real‐time feedback optimizes the synergistic effects of bracing and neuromuscular re‐education, potentially disrupting the “vicious cycle” of asymmetric loading and curve progression [16].

Notably, the tele‐rehabilitation group's lower progression rate (2.9% vs. 6.1%) mirrors findings from recent studies evaluating digital adherence monitoring in bracing therapy [17]. This supports the hypothesis that virtual supervision mitigates two key limitations of autonomous home exercise: progressive loss of corrective exercise form and declining compliance over time [18]. The earlier improvements in SRS‐22 domains (function and satisfaction) observed in the supervised cohort by 12 months further reinforce the temporal relationship between exercise fidelity and quality of life enhancements [19].

### 4.2 Mechanistic Implications

Three interrelated mechanisms may explain the tele‐rehabilitation cohort's superior outcomes:

1. Enhanced Neuromuscular Precision: Real‐time correction of rotational breathing patterns and core activation likely improved paravertebral muscle symmetry, augmenting the Chêneau brace's derotational forces. Electromyographic studies have established that improper exercise execution reduces muscular torque generation, suggesting that virtual supervision preserves therapeutic biomechanics [20].
2. Dynamic Adherence Reinforcement: The significantly higher compliance in the tele‐rehabilitation group (92.6% vs. 74.3%; *p* < 0.001) created a virtuous cycle: improved exercise fidelity → enhanced outcomes → increased motivation. This directly addresses the 58% attrition rate typical of unsupervised programs at 18 months [21].
3. Precision Load Modulation: Synchronized brace adjustments and exercise progression based on monthly radiographic reviews may have optimized growth modulation—a critical advantage given participants' skeletal immaturity (Risser ≤ 2) [22]. The differential ATR improvements (Δ4.0° vs. Δ2.0°, *p* < 0.01) particularly highlight tele‐rehabilitation's capacity to address axial plane deformities, which are often poorly controlled by bracing alone [23]. This aligns with emerging evidence that rotational correction requires coordinated brace‐exercise interactions rather than passive orthotic forces [24].

### 4.3 Clinical Translation

These findings carry immediate implications for AIS management paradigms:

1. Resource‐limited settings: Tele‐rehabilitation protocols could democratize access to SOSORT‐standard care in regions lacking specialized spinal therapists, potentially reducing global disparities in scoliosis outcomes.
2. Pandemic resilience: The protocol's success during extended healthcare disruptions (2020–2022) provides a template for maintaining continuity of care during future public health crises [25].
3. Precision rehabilitation: Integrating motion capture sensors (not utilized here) with existing telemedicine platforms could further personalize exercise prescriptions based on real‐time kinematic data. However, three caveats temper enthusiasm. First, the single‐center design and modest sample size (*n* = 67) limit generalizability to severe curves (Cobb > 45°) or older adolescents [26]. Second, while the widespread accessibility of Tencent Meeting in China supported smooth implementation, platform‐dependent protocols may face regulatory hurdles in other healthcare systems [27]. Finally, the 24‐month follow‐up precludes assessment of postmaturity curve rebound—a phenomenon affecting 15%–20% of braced patients [28].

### 4.4 Limitations

This study has several limitations:

1. Generalizability constraints due to the single‐center design and technology‐dependent protocol.
2. Outcomes were assessed up to skeletal immaturity (24 months). The durability of correction beyond this point, particularly at full maturity (Risser 5), remains unverified.
3. Due to the inherent nature of tele‐rehabilitation interventions, participants and therapists were not blinded to group allocation. This may introduce performance bias.
4. The SRS‐22 may exhibit ceiling effects in pain/satisfaction domains within conservative cohorts, potentially underestimating subtle quality of life changes.
5. While our study demonstrated the superiority of tele‐rehabilitation in improving Cobb angles and ATR, we did not evaluate advanced coronal imbalance metrics such as the Coronal Deformity Angular Ratio (C‐DAR). Recent evidence has established C‐DAR > 6 as an independent predictor of bracing failure (OR = 2.11) [29].

### 4.5 Future Directions

1. Future multi‐center trials should validate these findings across diverse healthcare systems and incorporate low‐tech alternatives to enhance accessibility.
2. Future studies should track cohort outcomes to skeletal maturity (Risser 5) to assess the durability of correction.
3. Future trials should implement blinded outcome assessors and incorporate objective biomechanical measures less susceptible to observer bias.
4. Future studies should integrate condition‐specific tools (e.g., Brace Questionnaire, BrQ) alongside the SRS‐22 to better capture psychosocial impacts and treatment burden.
5. Future comparative studies of PSSE delivery methods should incorporate C‐DAR to assess their interaction with coronal alignment.

## 5 Conclusion

This study establishes tele‐rehabilitation as a transformative adjunct to brace therapy, demonstrating statistically and clinically superior outcomes across radiographic, scoliometric, and patient‐reported metrics. By merging digital supervision with established conservative modalities, we advance toward personalized, equitable, and resilient AIS management systems. Future iterations incorporating artificial intelligence‐driven motion analysis may further revolutionize scoliosis care delivery, particularly for adolescents navigating the dual challenges of treatment adherence and developmental transitions.

## Author Contributions

All authors had full access to the data in the study and took responsibility for the integrity of the data and the accuracy of the data analysis. Conceptualization, T.C. and H.Z.; Methodology, L.C., Z.Z.; Investigation, Q.C.; Formal Analysis, Z.S.; Resources, X.W.; Writing – Original Draft, T.C.; Writing – Review and Editing, S.J.; Visualization, A.W.; Supervision, X.H. and A.W.

## Funding

This work was supported by the National Natural Science Foundation of China (Grant No. 82302783), the Zhejiang Provincial Health Commission Research Fund Project (No. WKJ‐ZJ‐26017), the Leading Disciplines of Zhejiang Province (Clinical Medicine, Wenzhou Medical University), the Wenzhou Major Science and Technology Innovation Project (No. ZY2023015), the High‐level Innovation Team of Wenzhou’s “Ouyue Talent Plan” (No. 2024R3003), and the Cross‐disciplinary Research Project of the Provincial Peak Discipline (Pharmacy) at Wenzhou Medical University (No. 437502516).

## Ethics Statement

This study was approved by the Institutional Review Board of the Second Affiliated Hospital and Yuying Children's Hospital of Wenzhou Medical University (LCKY2020‐295). Informed consent was obtained from all participants. All methods were performed following the relevant guidelines and regulations.

## Conflicts of Interest

The authors declare no conflicts of interest.

## Supporting information


**Data S1:** os70265‐sup‐0001‐Supinfo.docx.

## Data Availability

The data that support the findings of this study are available from the corresponding author upon reasonable request.
