# Supplementary material for: Effects of Bracing Combined With Tele‐Rehabilitation‐Guided Family Physiotherapeutic Scoliosis‐Specific Exercises on Adolescent Idiopathic Scoliosis
Source: Orthop Surg. 2026 Jan 30;18(3):511–22. doi: 10.1111/os.70265 (PMC12967554; doi:10.1111/os.70265)
Supplement: Supplementary file 1 — Data S1: os70265‐sup‐0001‐Supinfo.docx. [file OS-18-511-s001.docx]

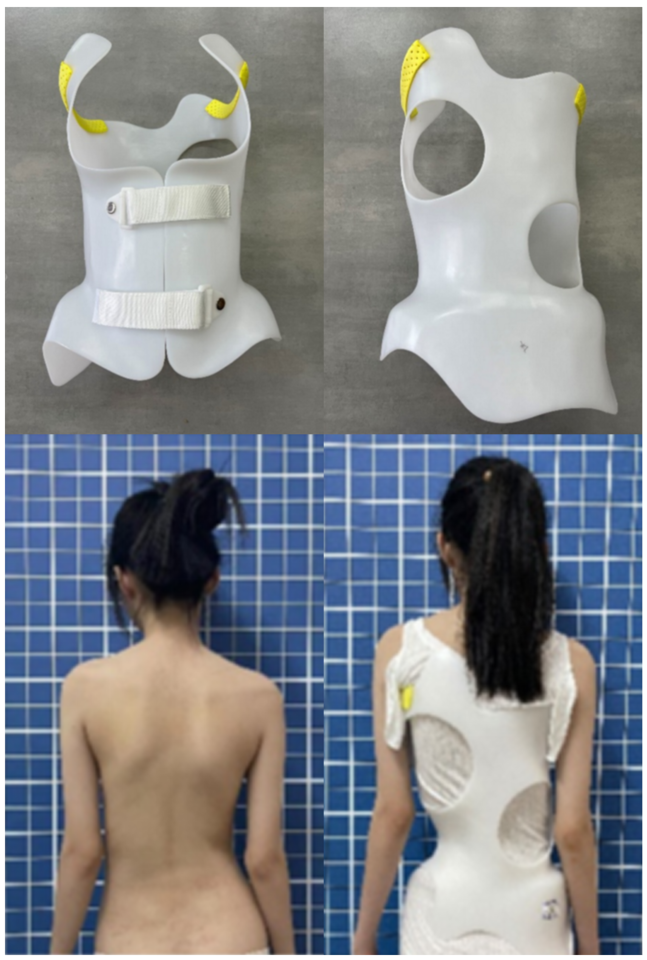


**Fig. S1** **Representative Chêneau brace manufactured for the study.**The custom orthosis was fabricated from 4 mm thick high-temperature polyethylene thermoplastic sheets.


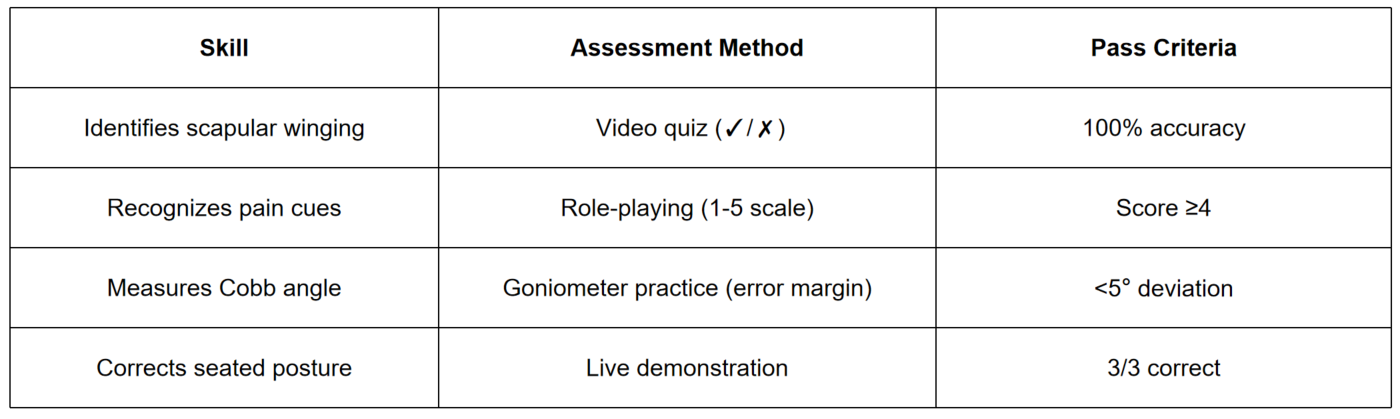


**Fig. S2** Guardian competency standards. Pre-training assessment protocol with pass/fail criteria for critical supervision skills: postural deviation identification (100% accuracy), pain recognition (≥4/5), angle measurement (<5° error), and posture correction (3/3 demonstrations correct).


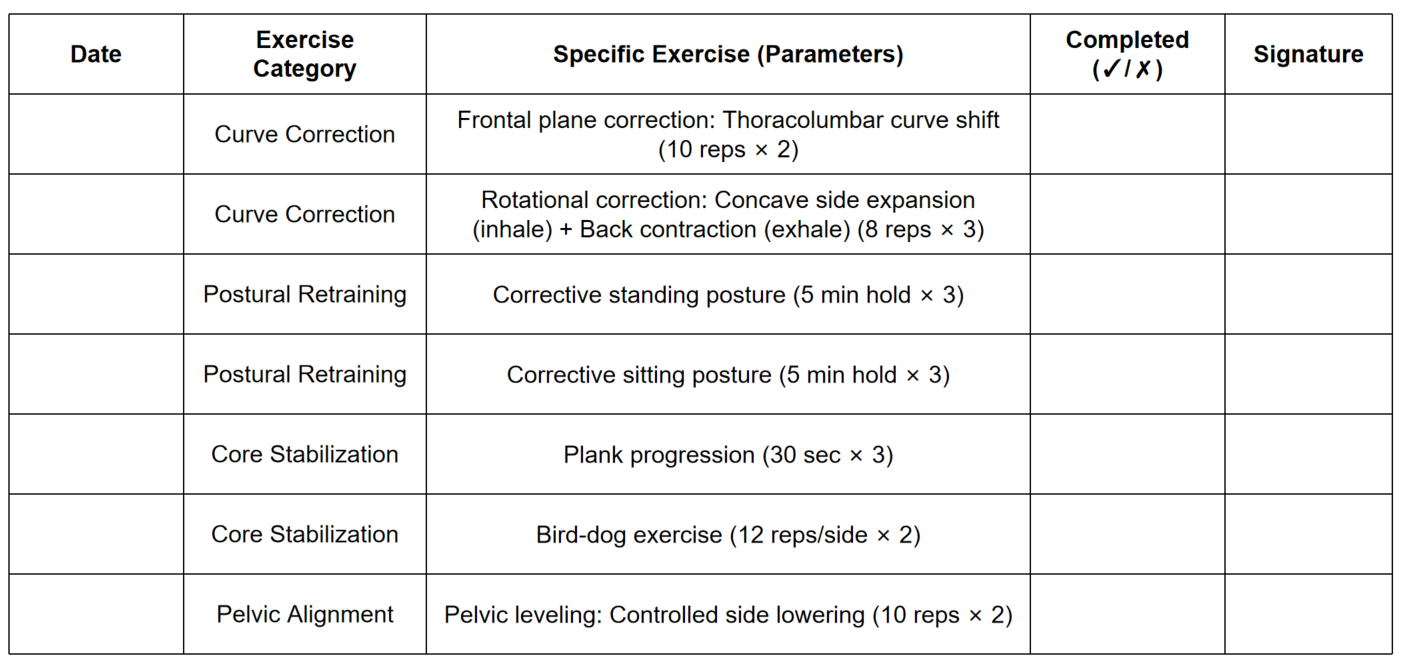


**Fig. S3** Universal scoliosis exercise log template. This direction-free format maintains standardized exercise parameters aligned with Fig. 1 interventions while allowing therapists to apply personalized directional instructions based on individual patient presentations.
